# A broadly adaptable protocol for isolating Kupffer cells from non-model species: application to *Mastomys natalensis* and its susceptibility to Old World mammarenaviruses

**DOI:** 10.3389/fimmu.2025.1713374

**Published:** 2025-12-04

**Authors:** Nicolas Corrales, Katharina Hansen-Kant, Angelika Lander, Joseph B. Prescott

**Affiliations:** Center for Biological Threats and Special Pathogens, Robert Koch-Institut, Berlin, Germany

**Keywords:** Kupffer cell, non-model species, *Mastomys natalensis*, mammarenavirus, comparative immunology, reservoir host, primary macrophages

## Abstract

**Background:**

Kupffer cells are specialized, liver-resident macrophages that play central roles in hepatic homeostasis, immune surveillance and pathogen clearance. While well-studied in laboratory mice and rats, methods for isolating and studying Kupffer cells from non-model species remain scarce, limiting research in ecologically and zoonotically relevant hosts such as the Natal multimammate mouse (*Mastomys natalensis*), the natural reservoir of Lassa virus (LASV).

**Methodology and principal findings:**

We developed and validated an optimized Kupffer cell isolation protocol adaptable to non-model rodents, relying on mechanical and enzymatic liver dissociation, non-parenchymal cell enrichment by Percoll gradient and selection by adherence. Critical parameters affecting yield and viability included maintaining all perfusion and digestion steps at 37 °C, limiting enzymatic digestion to ≤15min and avoiding aggressive mechanical disruption. Under optimized conditions, yields averaged 2.55 ± 1.13 × 10^5^ viable Kupffer cells per gram of liver (≈80% viability). Isolated cells displayed macrophage-like morphology, expressed a Kupffer cell marker profile (CD11b^+^/Iba1^+^/MHC-II^+^/CD80^+^) and demonstrated phagocytic and pinocytic activity. As proof-of-concept, Kupffer cells were infected *in vitro* with LASV or Mopeia virus (MOPV). Both *Mammarenaviruses* successfully infected Kupffer cells, but infection kinetics differed; LASV persisted at stable levels without cytopathic effect, whereas MOPV replication declined over time, suggesting virus-specific control mechanisms.

**Conclusions and significance:**

This protocol provides a robust, reproducible, and species-flexible method for isolating viable Kupffer cells from non-model rodents without requiring species-specific reagents. It enables functional, phenotypic, and virological studies in primary hepatic macrophages from *M. natalensis* and can be adapted to other wildlife reservoirs, supporting comparative immunology, zoonotic disease ecology, and host–pathogen interaction research in under-characterized species.

## 1 Introduction

The liver is a unique immunological organ that acts as both a metabolic hub and a critical immune interface. Constantly exposed to blood from the gastrointestinal tract, the liver must balance immune tolerance toward harmless dietary and microbial antigens with effective responses to pathogens and tissue damage. This dual function is orchestrated by a complex interplay of parenchymal cells (such as hepatocytes) and non-parenchymal cells —including sinusoidal endothelial cells, hepatic stellate cells, lymphocytes and resident macrophages, known as Kupffer cells (1, 2).

Kupffer cells are the largest population of tissue-resident macrophages in the body and are strategically embedded in hepatic sinusoids, where they capture blood-borne particles and microorganisms. Unlike macrophage populations that are replenished from circulating monocytes, Kupffer cells arise during embryonic development and self-renew *in situ (*3). This distinct origin, combined with their continuous exposure to microbial and metabolic signals, endows them with a unique transcriptional profile and functional specialization that differs from other tissue-resident macrophages (4). Kupffer cells play key roles in steady-state immune regulation, pathogen clearance, and the initiation of immune responses during infection, cancer, or liver injury (3).

Given their central role at the crossroads of metabolism and immunity, Kupffer cells have become a subject of intense interest across diverse fields, from hepatology and immunology to virology and toxicology (5, 6). In particular, their contribution to disease outcomes during liver infections, autoimmunity, and systemic inflammatory conditions is increasingly recognized (7, 8). However, research on Kupffer cell biology has largely relied on laboratory mice and rats, leaving a gap in our ability to study these cells in non-model species, including those of ecological, zoonotic or translational importance.

This challenge is especially acute when studying wildlife reservoirs of emerging pathogens, where species-specific tools such as antibodies or cytokines are lacking (9). A standardized, adaptable protocol for the isolation and characterization of Kupffer cells that minimizes reliance on species-specific reagents would greatly advance comparative immunological studies in non-model organisms.

Here, we present an optimized Kupffer cell isolation protocol based on mechanical and enzymatic liver dissociation, non-parenchymal cell enrichment and selection by adherence. Importantly, the method is compatible with commonly used cross-reactive reagents and does not rely on species-specific antibodies or genetically characterized animals. We identify critical steps that affect yield and viability and validate the method in the Natal multimammate mouse (*Mastomys natalensis*), an African rodent of high zoonotic relevance.

The Natal multimammate mouse is the natural reservoir of Lassa virus (LASV), a zoonotic hemorrhagic fever virus endemic to West Africa, along with several other related viruses. Unlike humans, the natal mouse displays only minor clinical signs or liver pathology upon LASV infection, making it an important system for studying tolerance to viral infection in natural hosts (10, 11). Using our protocol, we successfully isolated viable Kupffer cells from natal mice livers, confirmed their identity and functionality and demonstrated their permissiveness to arenavirus infection.

This method establishes a flexible platform for Kupffer cell isolation from non-model rodents that can be applied to other mammalian species, enabling new opportunities for virological, immunological, and ecological studies in reservoir hosts, wildlife models and under-characterized animals.

## 2 Results

### 2.1 Identification and optimization of key steps for Kupffer cell recovery

Given the desire to isolate viable Kupffer cells from natal multimammate mice, we initially used a standard protocol for Kupffer cell isolation from laboratory mice (*Mus musculus*) *(*5). However, early attempts at following this protocol yielded poor Kupffer cell recovery and inconsistent cell viability, suggesting that the original procedure was not directly generalizable for all rodents. To improve outcomes, we performed a stepwise evaluation the variables, modifying each parameter serially, to determine their impact on Kupffer cell recovery and/or viability; namely: 1) perfusion medium temperature, 2) digestion medium temperature, 3) digestion time, 4) presence of growth factors during the procedure and 5) method of mechanical dissociation of the liver.

**Figure 1:**
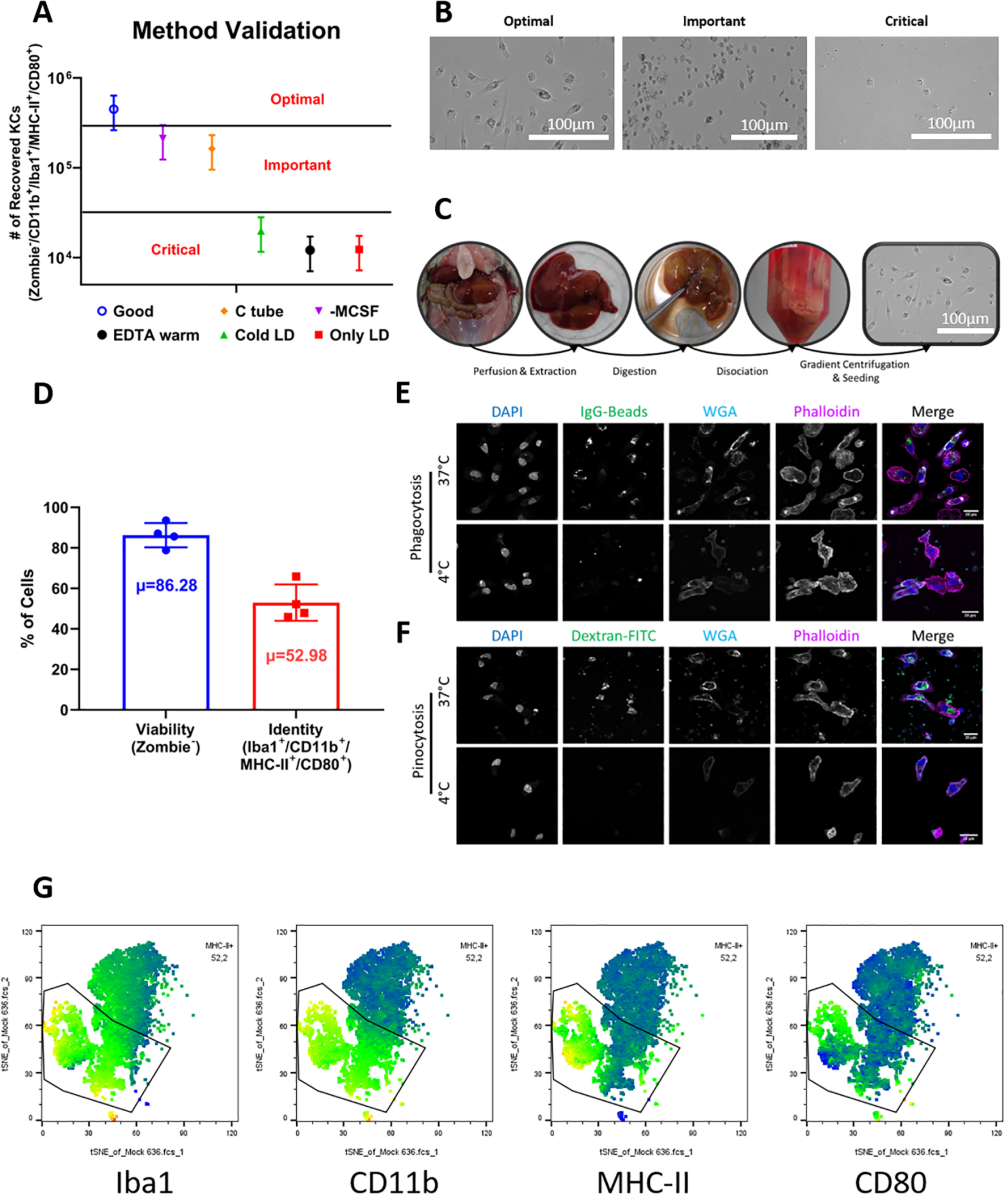
Optimization, validation, and characterization of Kupffer cell isolation from natal multimammate mice. **(A)** Yield of Kupffer cells obtained under different protocol conditions. Cell numbers were determined by flow cytometry with counting beads. Bars represent mean ± SD from four biological replicates for the optimized protocol. To adhere to 3R principles and avoid unnecessary animal use, the other experimental conditions were tested in single animals; their error ranges were extrapolated using the coefficient of variation (CV) derived from the optimized group to illustrate data dispersion, but were not included in statistical analyses. **(B)** Brightfield microscopy illustrating Kupffer cell morphology under different conditions. Under optimal conditions, cells appeared abundant and displayed the characteristic spread macrophage-like morphology; under “important” conditions, yields were reduced but adherent cells retained typical morphology; under “critical” conditions, cells were sparse and showed poor adherence or abnormal shape. **(C)** Documentation of the main protocol steps, showing macroscopic changes in liver tissue during perfusion, digestion, dissociation, and seeding. **(D)** Flow cytometry analysis of cell viability (Zombie^-^) and Kupffer cell phenotype (Iba1^+^/CD11b^+^/MHC-II^+^/CD80^+^) among viable cells. Data shown as mean ± SD from four animals; µ indicates the average marker frequency. **(E)** Phagocytic activity of isolated Kupffer cells assessed by confocal microscopy using fluorescent IgG-coated particles. Uptake is shown for cells incubated at 37 °C or at 4 °C as negative control. **(F)** Pinocytic activity assessed using fluorescent dextran under the same temperature conditions. Confocal images are representative of three independent experiments. Cell nuclei were stained with DAPI (blue), membranes with WGA (cyan), and actin cytoskeleton with phalloidin (magenta). Merged images were processed using ImageJ. **(G)** Representative tSNE plots from flow cytometric analysis. Each plot displays the mean fluorescence intensity (MFI) of a single marker, with the Kupffer cell population (Iba1^+^/CD11b^+^/MHC-II^+^/CD80^+^) highlighted.

Kupffer cell yield was quantified by flow cytometry using counting beads. We observed that even minor deviations from the prescribed buffer temperatures and enzyme preparation significantly affected the efficiency of the procedure, with the optimal combination of variables producing the highest number of viable adherent cells (**Figure 1A**). Representative brightfield images support these observations, showing increasingly compromised cell morphology when deviating from optimal conditions. Cell morphology was especially affected under conditions involving perfusion with cold buffers, prolonged digestion, or mechanical dissociation via shredding techniques (**Figure 1B**).

We categorized the tested variables into three groups: optimal, important, and critical, based on the resulting number and quality of recovered Kupffer cells. The variables deemed critical for maintaining high viability and yield were:

1. performing liver perfusion with EDTA solution maintained strictly at 37 °C,
2. ensuring full pre-warming and solubilization of the Liver Digest solution to 37 °C before its use, and
3. avoiding prolonged incubation of the livers in the Liver Digest solution by ensuring that the total exposure time does not exceed 15 minutes, and promptly transferring the tissue into culture medium before dissociation.

When these conditions were not maintained, there was a reduction in the number of recovered cells and a visible loss of morphological integrity (**Figures 1A, B**, “Critical”).

Two other variables were found to be important, though not strictly critical (**Figures 1A, B**, “Important”):

1. the presence of 50ng/mL of recombinant macrophage colony-stimulating factor (M-CSF) during the resuspension of liver non-parenchymal cells, and
2. the method of mechanical dissociation. Cells resuspended in the absence of M-CSF still adhered to tissue culture plates but showed signs of stress, including reduced flattening and failure to develop the characteristic elongated macrophage-like morphology, as well as increased fragility. Additionally, mechanical dissociation using Miltenyi C-tubes induced more extensive cell damage than dissociation by gentle agitation in culture medium.

These findings provided a clear framework for defining a robust, reproducible, and gentle protocol adapted specifically for this non-model species.

Based on these optimization criteria, we established a streamlined and reproducible protocol tailored for Natal mouse livers. The procedure follows four major steps—perfusion, digestion, dissociation, and gradient centrifugation followed by cell seeding in tissue culture plates (**Figure 1C**). A key advantage of this protocol is that it allows ex vivo perfusion of the liver, leaving the remaining carcass and the hepatocyte fraction of the liver intact, and thus potentially available for other downstream applications. Additionally, only a portion of the liver (e.g., a single lobe) needs to be perfused and digested to obtain sufficient cell numbers in many cases, allowing the remaining intact liver tissue to be possibly used for parallel histological, transcriptomic or proteomic analyses from the same animal.

Using this optimized protocol, we consistently obtained 1.43-3.97 × 10^5^ Kupffer cells per gram of liver tissue, with an average yield of 2.55 ± 1.13 × 10^5^ cells/g (N = 4). These values were derived from livers ranging from 4.4 to 6.4g in mass (**Table 1**). Cell viability was approximately 80%, assessed by dye exclusion the day after isolation (**Figure 1D**), indicating minimal cytotoxicity during preparation. The adherent cells displayed a consistent macrophage-like morphology across all animals and replicates, with flattened, spread-out shapes indicative of resting macrophages (**Figure 1C**, rightmost image).

**Table 1:** Yield and recovery efficiency of Kupffer cells isolated from *Mastomys natalensis* livers.

Subject	Liver mass (g)	LNPCs (x10^6^)	Recovered Kupffer Cells (x10^5^)	% of Recovery
1	6.4	17.50	9.12	5.21
2	4.6	18.76	13.44	7.16
3	4.4	14.00	8.32	5.94
4	5.6	27.30	22.24	8.15
Average	5.25	19.39	13.28	6.62
SD	0.81	4.89	5.53	1.13
%CV	15.43	25.22	41.64	17.07

To further confirm the identity of the isolated cells as Kupffer cells, we performed both functional and phenotypic characterization. Confocal microscopy was used to evaluate phagocytic and pinocytic activity. As expected for Kupffer cells, isolated cells showed robust uptake of IgG-coated fluorescent beads and fluorescent dextran at 37 °C, while uptake was negated at 4 °C, confirming the specificity of the phagocytosis process and the metabolic activity of the cells (**Figures 1E, F**).

Phenotypic analysis by flow cytometry revealed that approximately 53% of viable cells expressed a surface marker profile consistent with Kupffer cells, defined as CD11b^+^/Iba1^+^/MHC-II^+^/CD80^+^ (**Figure 1D**). This marker combination allowed us to distinguish Kupffer cells from potential contaminating populations in the liver non-parenchymal cell fraction (**Figure 1G**), and the consistency of marker expression across replicates further supports the reliability of the protocol (**Figure 1D**).

Taken together, these data demonstrate that small but deliberate adjustments to a pre-existing Kupffer cell isolation protocol can yield high-quality, viable liver-resident macrophages from Natal mouse.

### 2.2 Kupffer cells from the Natal multimammate mouse are permissive to LASV and MOPV infection

A central goal in developing this isolation protocol was to enable functional studies on Kupffer cells from non-model species. To test the suitability of our protocol for such downstream applications, we performed a proof-of-concept infection experiment using our optimized Kupffer cell isolation method.

**Figure 2:**
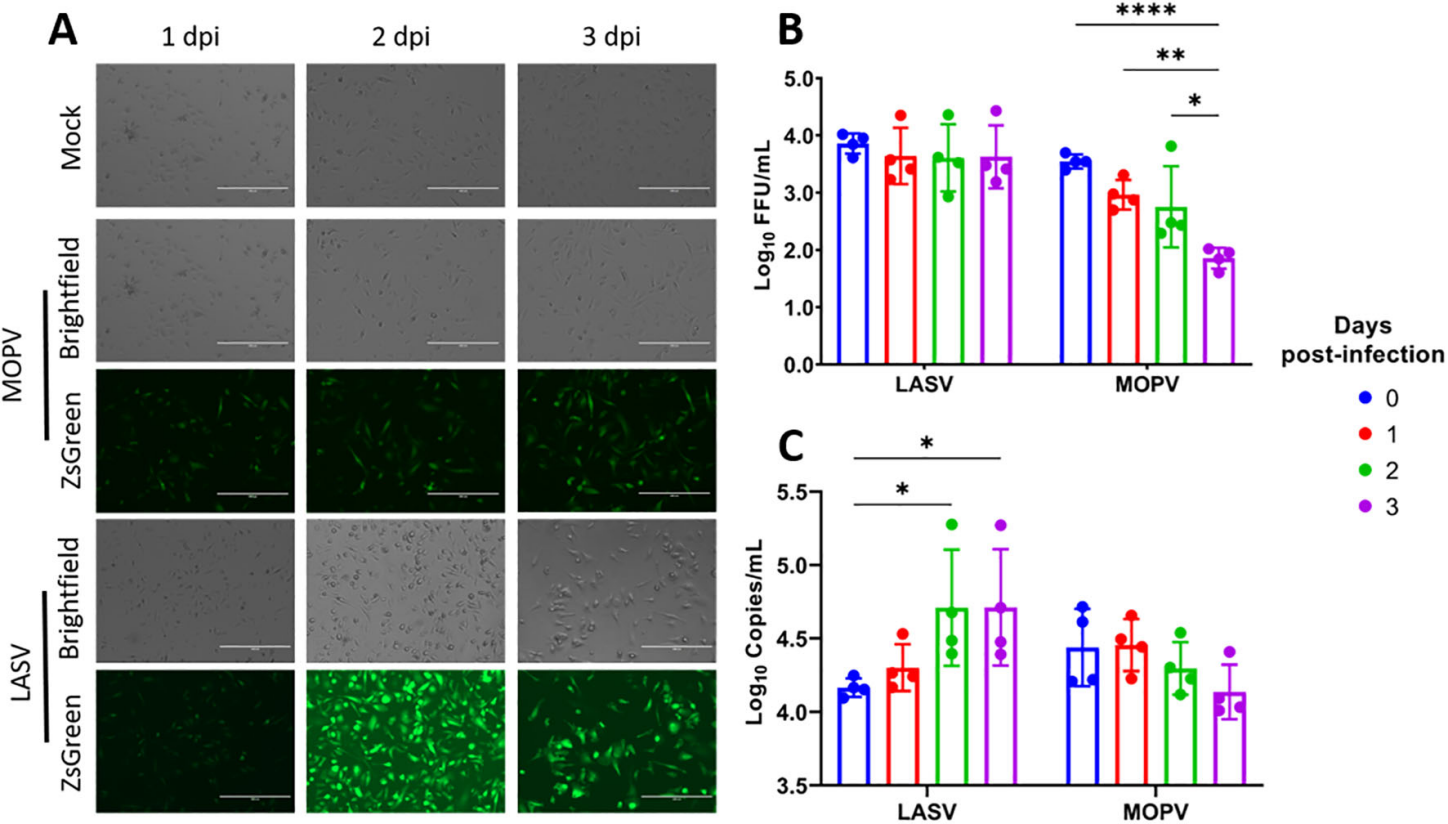
Kupffer cells from the natal multimammate mice support infection by LASV and MOPV. **(A)** Representative brightfield and fluorescence microscopy images of Kupffer cells infected with Mock, MOPV-ZsGreen or LASV-ZsGreen from 1-3dpi. **(B)** Virus quantitation in the supernatant from LASV- and MOPV-infected cells measured by focus-forming assay from day 0–3 dpi. **(C)** Viral RNA levels in the supernatant quantified by RT-qPCR for LASV and MOPV from day 0–3 dpi. Statistical analysis was performed using two-way ANOVA with Dunnett’s post hoc test. ****p<0.0001; **p<0.01; *p<0.05. The scale bar on the microscopy pictures represent 200μm.

Kupffer cells were isolated from Natal mouse livers using our optimized protocol and seeded in tissue culture plates for infection the following day. Cells were infected with LASV-ZsGreen or MOPV-ZsGreen. Uninfected (Mock) Kupffer cells served as a baseline. Fluorescent and brightfield microscopy were used to visually monitor the cultures at 1, 2, and 3 days post-infection (dpi). Both LASV-ZsGreen and MOPV-ZsGreen showed detectable fluorescence in infected cultures by 1 dpi, indicating successful viral entry and viral protein expression. This signal persisted through 3 dpi without observable decline in intensity, suggesting sustained infection and replication (**Figure 2A**).

To assess the production of viable viral progeny, we collected supernatants at 24 h intervals over three days and measured viral load by both RT-qPCR targeting the nucleoprotein (NP) gene and infectious titer using a focus-forming assay. The results demonstrated that Kupffer cells from the Natal mice are permissive to infection by both LASV-ZsGreen and MOPV-ZsGreen, although the replication kinetics differed between the two viruses. In the case of LASV, we observed that infectious titers remained stable, with no significant increase from baseline (**Figure 2B**). However, we saw a steady and significant increase in NP-gene copies in the supernatant over time, consistent with active viral replication, suggesting that viral RNA was being produced but may not have been fully translated into infectious progeny under these conditions (**Figure 2C**). In contrast, MOPV-infected cultures showed a declining trend in both NP-gene copies and infectious titers over the 3-day period.

In summary, Kupffer cells isolated with this protocol could be maintained in culture, displayed stable morphology, and supported infection with both LASV and MOPV. While LASV infection was associated with sustained levels of viral RNA, MOPV infection showed a decline in replication over time, indicating distinct replication dynamics between the two viruses.

## 3 Methods

### 3.1 Ethics statement

Natal Multimammate mouse samples were obtained according to the procedures approved by ethics committee of the Berlin state’s office for Health and Social (LAGeSO Berlin) and the Robert-Koch Institute (agreement TN0001/22).

### 3.2 Kupffer cells isolation

#### 3.2.1 Liver perfusion

Liver Digest solution (Gibco) and HBSS (Gibco) supplemented with 0.5mM Na_2_EDTA (Merk) were brought and maintained at 37 °C for the duration of the procedure. Animals were euthanized according to approved methods (isofluorane anesthesia, followed up by cardiac puncture and cervical spinal dislocation). Immediately after, the abdominal cavity was opened and the liver field cleared for identification of the inferior cava vein. The cava vein was cannulated with a 25G needle and 200µL of room-temperature enoxaparin solution (500Units/mL; Clexane^®^) was administrated using a 1mL syringe (Omnifix-F, Braun). The portal vein was severed, and liver perfusion was initiated using a 10mL syringe (Braun) filled with pre-warmed HBSS-EDTA, administered at a steady rate of approximately 5mL/min to clear residual blood and begin disrupting cell-to-cell contacts. A second syringe of pre-warmed HBSS-EDTA was prepared and kept warm at 37 °C in a warm bath, so immediately after completing the first syringe, the second 10mL syringe was used to deliver an additional 5mL of solution. The remaining 5mL in the second syringe were kept in reserve in case of technical issues during perfusion and discarded if not required.

#### 3.2.2 Liver excision and digestion

Livers were carefully excised and transferred to a sterile Petri dish. Warm Liver Digest Medium (37 °C) was slowly and gently injected into the peripheral regions of each liver lobe, directing the flow from the outer parenchyma toward central vascular structures such as the portal vein and vena cava. Perfusion was performed using 10mL syringes at a controlled rate of approximately 3mL/min, ensuring gradual diffusion of the enzyme solution without mechanical disruption of the tissue. The administration proceeded in phases. In the initial phase, a full 10mL of Liver Digest Medium was administered, after which excess fluid was expelled through the liver capillaries and tissue pores. This initial outflow, containing blood residues and digested cellular debris, was discarded, and the liver was transferred to a fresh Petri dish to prevent contamination. An additional 10mL aliquots of Liver Digest Medium were administered continuing from the periphery toward the center of each lobe, while carefully observing the progression of tissue digestion – as described below.

The extent of dissociation was assessed visually throughout the process. Indicators of sufficient digestion included a shift in liver color to pale beige, softening of the lobes and detachment of parenchymal tissue from the liver capsule (**Figure 1C**). If these features were observed before full administration of the second or third syringe, perfusion was paused and the liver was allowed to incubate undisturbed for the remainder of the time. Critically, the total digestion time did not exceed 15 minutes from the first injection and the maximum total volume of Liver Digest Medium administered per liver was limited to 45mL to prevent over-digestion and cell damage.

To terminate the digestion, the liver lobes were gently transferred to a 50mL conical tube (TPP). The remaining fluid in the Petri dish, now containing released Kupffer cells, was carefully collected using a serological pipette and added to the same tube. The tube was then supplemented with 5mL of cold RPMI 1640 medium (Sigma), supplemented with 100U/mL penicillin, 100μg/mL streptomycin (Gibco), 2mM L-glutamine (Gibco), 1% HEPES (Gibco), and 10% FCS (Biochrom) and immediately placed on ice. At this point, samples could either be processed directly or transported between facilities (e.g., from a manipulation room to a cell culture laboratory).

#### 3.2.3 Liver dissociation

With the digested liver sections in a 50mL conical tube containing ice-cold RPMI medium (as described above), using sterile traumatic-tipped surgical forceps, the liver section was gently held by the central connective tissue joining the lobes. Under sterile cell culture conditions and by visual inspection, a second pair of forceps was used to make several shallow tears along the surface of each liver lobe to initiate decapsulation. These small tears help to loosen the outer capsule and allow underlying dissociated parenchyma to efficiently disperse.

Immediately following the capsule disruption, the liver capsule was gently held with forceps and manually agitated within the same 50mL tube containing RPMI medium. The tissue was swirled and shaken by hand for several seconds at a time, alternating with brief visual inspections. As the capsule began to detach and the parenchyma broke down further, loosened pieces of capsule were manually removed using the forceps. This process continued for approximately 5 minutes per liver until most of the parenchymal contents had formed a cloudy suspension.

At the end of this step, the liver capsule was largely intact and free of visible parenchymal tissue, minimizing contamination from extracellular matrix debris. The resulting cell suspension contained: hepatocytes, parenchymal cells and the liberated non-parenchymal fraction - including Kupffer cells - along with occasional larger tissue fragments. No filtration step was applied at this stage; the entire content of the tube proceeded directly to the next centrifugation step.

#### 3.2.4 Isolation of liver non-parenchymal cells

The 50mL tube containing the dissociated liver tissue was centrifuged at 20 × g for 3 minutes at 4 °C. This low-speed spin pelleted primarily hepatocytes, while the non-parenchymal cells remained in suspended. The supernatant was carefully transferred to a new 50mL tube by gentle inversion, and the centrifugation step was repeated to further reduce hepatocyte contamination.

The resulting non-parenchymal cells containing supernatant was then mixed thoroughly by inversion at a 1:2 ratio with a 36% Percoll solution (Cytiva, diluted in distilled water). This mixture was centrifuged at 850 × g for 20 minutes at room temperature, without a brake. The resulting pellet contained the enriched liver non-parenchymal cells.

In cases where erythrocyte contamination was visible, the pellet was resuspended in ACK lysis buffer (Gibco) following the manufacturer’s protocol (1mL for 5 minutes at room temperature), then centrifuged at 850 x g for 5 min at 4 °C and the supernatant was discarded. The pellet was kept for the further steps.

#### 3.2.5 Selection of Kupffer cells by adherence

The resulting liver non-parenchymal cells were resuspended in RPMI 1640 (Sigma) supplemented with 50ng/mL of mouse recombinant M-CSF (R&D Systems), 100U/mL penicillin, 100µg/mL streptomycin (Gibco), 2mM L-glutamine (Gibco), 1% HEPES (Gibco) and 10% FCS (Biochrom). Then they were filtered through a 40µm cell strainer and plated at a density of maximum 1x10^7^ liver non-parenchymal cells (LNPCs) per well in a 6-well plate using 1mL of the same medium.

After 2h of incubation at 37 °C and 5% CO_2_, non-adherent cells were removed by removing the medium and gently washing the adherent cell layer with cold PBS. The remaining attached cells were incubated in 3mL of fresh medium and used for further experiments and characterization following day.

### 3.3 Confocal microscopy

For confocal fluorescence microscopy imaging, cells were stained with wheat germ agglutinin (WGA)-AleaFluor594 (Invitrogen), fixed with 10% formalin (HistoFix, Roth) and then stained with Acti-Stain 670 (Cytoskeleton) and DAPI (RotiMount, Roth). All according to manufacturer’s instructions. Imaging was performed using the Stellaris 8 confocal microscope (LEICA) from the unit for Advanced Light and Electron Microscopy at the Center for Biological Threats and Special Pathogens from the Robert Koch Institute. Image processing was performed using ImageJ software (12).

### 3.4 Phagocytosis and pinocytosis assays

Phagocytosis analysis with FITC-beads was performed according to manufacturer instructions (Cayman-Chem). After 2 h of incubation at 37 °C with FITC-IgG-beads, the cells were kept permanently on ice to be microscopically or flow cytometrically analyzed. Signals of extracellular non-internalized beads were quenched by addition of Trypan blue (0.04%, Lonza) for 2–3 min and two subsequent washes with cold PBS prior to measurements. Pinocytosis was measured as the intracellular absorption of soluble FITC-Dextran (Sigma-Aldrich; 40,000 Da, final conc. 0.5 mg/mL) after 2 h of incubation at 37 °C. Free unbound FITC-Dextrane was removed by two washes with ice-cold PBS prior to measurements, and stainings were performed as described above.

### 3.5 Flow cytometry

For flow cytometric analysis, the cells were harvested and first incubated with Fc-block buffer (BioRad #MCA2305) for 15 mins. After that, the cells were incubated with antibodies against CD11b (Biolegend #101216, 1:200), MHC-II (Invitrogen #12-5980-82, 1:200), CD80 (Biolegend #104713, 1:200), Iba1 (Fujifilm #019-19741, 1:50) and ZombieNIR (Biolegend #423106, 1:200) for 15 min at room temperature in the dark. The samples were washed 3 times with FACS buffer (PBS 1% BSA, 0.1% NaN3) and then fixed overnight with 10% formalin (HistoFix, Roth). After that, the cells were centrifuged at 1000 x g for 10 min, and resuspended in FACS buffer and Precision Counting Beads (Biolegend #424902, 1:2). After that, the cells were directly analyzed.

All flow cytometric measurements were performed on a Cytoflex S (Beckmann Coulter). The data was graphed and analyzed using FlowJo™ v10.8 Software (BD Life Sciences), using single color and fluorescence-minus-one controls for compensation, and isotype controls for thresholding, and GraphPad Prism v10 for Windows (Boston, Massachusetts USA) software.

### 3.6 Statistical analysis

All statistical analyses were performed using GraphPad Prism v10 for Windows. Unless otherwise indicated, results are expressed as mean ± standard deviation (SD). In **Figure 1A**, four biological replicates (N = 4) were performed for the optimized protocol, while single animals were used for the remaining conditions to minimize animal use in accordance with the 3R principles. To visualize data dispersion for these single-replicate conditions, the coefficient of variation (CV) obtained from the optimized protocol replicates was applied to the corresponding mean values to generate extrapolated SDs. These extrapolated values were used solely for graphical representation and were not included in the formal statistical testing, which was restricted to conditions with true biological replication. For **Figure 2**, one-way analysis of variance (ANOVA) followed by Dunnett’s *post hoc* test was used.

### 3.7 Biosafety

All experiments with Risk Group-4 viruses were conducted under maximum containment conditions in the biosafety level-4 (BSL4) laboratory of the Robert Koch Institute, in accordance with standard operating procedures institutionally approved by the Robert Koch Institute.

### 3.8 Viruses

Recombinant LASV-ZsGreen was previously generated, characterized and kindly provided by Dr. Albariño from the CDC (13). Recombinant MOPV-ZsGreen was previously generated, characterized and kindly provided by Dr. Oestereich from BNITM (14). All Mammarenaviruses were propagated on Vero E6 cells (ATCC #CRL-1586) using DMEM (Sigma) supplemented with 100U/mL penicillin, 100μg/mL streptomycin (Gibco), 2mM L-glutamine, (Gibco) and 2% FCS (Biochrom).

### 3.9 Infection and sample harvesting

For *Mammarenaviruses*, the cells isolated from 4 animals were infected at a MOI of 2 for 1h, after which the medium was replaced by fresh medium and the cells were cultured for 3 days. Supernatant samples were collected every 24h for viral quantification.

### 3.10 Virus quantification

For viral quantification, supernatants were collected and titrated on Vero E6 cells. After 1h of infection, overlay medium (previously described DMEM + 1.5% carboxymethylcellulose) was added and cells were incubated for 5 days at 37 °C. After that, overlay medium was removed and the cells were fixed overnight with 10% formalin (HistoFix, Roth) for the inactivation of the virus. The fluorescent focus units were directly counted on a fluorescence microscope.

For viral copy numbers quantification, supernatants were inactivated using AVL buffer (Qiagen) and 70% ethanol, and then extracted using the QIAamp Viral RNA kit (Qiagen). The quantitative RT-PCR was performed using the primer-probe sets and protocol previously described (10).

## 4 Discussion

Molecular and immunological studies of non-model species have been an alluring challenge, given the valuable information we could obtain but also because of the lack of molecular species-specific tools (annotated genome sequences, antibodies, etc.) (9). Thus, the desire to establish and validate protocols for studies that don’t require species-specific reagents.

Herein, we describe an efficient protocol for the isolation and characterization of viable and functional Kupffer cells from Natal multimammate mice. The method does not require species-specific reagents for the isolation process itself. While cross-reactive antibodies and recombinant M-CSF were used here for quality control and maintenance of cell viability, other validation approaches (e.g., qRT-PCR or transcriptomic profiling) could be employed in their place. Given its flexibility and minimal reagent requirements, this protocol is likely adaptable to other non-model rodents and potentially to additional mammalian reservoir species. In particular, other rodent species and members of the Chiroptera order, such as various bat species known to harbor highly pathogenic VHFs, represent a compelling group for future adaptation of this method due to their ecological relevance (15).

The efficiency of our adapted protocol is consistent with that reported in for mice, where 1–3 x 10^6^ cells per harvested organ were obtained (5, 6). We were able to confirm the identity of the cells by surface marker and functional characterization. The cells showed a morphology consistent to macrophages (16). The surface expression of certain markers, allowed us to confirm CD11b^+^/Iba1^+^/MHC-II^+^/CD80^+^ cells as Kupffer cells (7, 17). Moreover, the cells displayed phago- and pinocytic activities, confirming the dynamic behavior associated with professional antigen presenting cells (18). While approximately 53% of the isolated cells displayed the already named surface marker combination, we acknowledge that the remaining cell population remains uncharacterized. Further phenotypic refinement of these fractions would be highly desirable; however, the current lack of validated cross-reactive antibodies for this non-model species poses a major limitation to such analyses. Nonetheless, based on the known cellular composition of LNPCs, which in other rodents typically includes about 50% sinusoidal endothelial cells, 25% lymphocytes, 5% biliary cells and 1% hepatic stellate cells (19, 20); the enrichment of Kupffer cells to over half of the culture fraction indicates a strong positive selection achieved by our method. Importantly, many of these potential “contaminant” cell types are themselves integral components of the hepatic microenvironment and actively contribute to innate immune regulation and inflammatory signaling (21). Their presence may therefore help preserve aspects of the physiological context in which Kupffer cells normally operate. Given the short duration of our infection assays, adaptive immune contributions are unlikely, and the observed responses can be interpreted as reflecting innate immune mechanisms within a Kupffer cell–enriched, yet microenvironmentally relevant, culture system.

We identified critical steps in this newly-adapted protocol, the reduction of the viable cell recovery yield is likely attributed to stress and disruption of hepatocytes during the sample preparation. On the one hand, hepatocytes have proven to be hard to culture *ex vivo* due to low viability and dedifferentiation processes occurring after harvesting (22). Conversely, these cells are known to express a wide variety of enzymes associated to biotransformation of molecules (RedOx catalysis mostly) (1) and a repertoire of proinflammatory cytokines (2). Both factors can be released to the Kupffer cell environment upon death of hepatocytes during the sample preparation and thus inducing a significant stress to the Kupffer cells. This would explain why the four most critical steps are associated to mechanical or enzymatic cell disruption due to over digestion, solute formation or mechanical shredding of the liver. Regarding the presence of the M-CSF in the media, this cytokine has been widely described as an inducer of survival and proliferation of macrophages by inducing the overexpression of the antiapoptotic protein Bcl-xL through the control of glucose uptake by the cells (23, 24). Thus, the presence of this cytokine might aid the stressed Kupffer cells recover and withstand the proinflammatory stimuli they get from damaged hepatocytes.

The primary motivation behind developing and validating this method was to enable future studies on the role of Kupffer cells in the apathogenic immune response to Mammarenavirus infection in their natural reservoir. The Natal multimammate mouse, is the natural reservoir host of Lassa virus (LASV) and closely related Mammarenaviruses such as Mopeia virus (MOPV) (11, 14). Interestingly, while both viruses are maintained in Natal mice populations, only LASV is known to be pathogenic in humans (14, 25, 26). Understanding how host-reservoir Kupffer cells interact with these viruses may provide insight into mechanisms of disease resistance and viral persistence. Previous reports demonstrated that, following experimental LASV infection of Natal mice, high viral titers can be detected in the liver. However, despite the presence of virus, little sign of hepatic tissue injury or immune cell infiltration are observed (10, 11). Moreover, viral titers in adult animals decline over time, and the infection is ultimately cleared. These findings are consistent with observations in human monocyte-derived macrophages infected with LASV, which also display a largely silent immune response (27). In contrast, the Kupffer cells isolated and infected in our study failed to control LASV replication but were capable of suppressing MOPV replication, indicating a virus-specific difference in their ability to respond.

This contrasting replication dynamics likely reflect differences in how each virus interacts with host cell-intrinsic antiviral mechanisms. The accumulation of LASV RNA in the absence of increasing infectivity may indicate the production of defective or non-infectious particles, as described for other RNA viruses such as influenza A and vesicular stomatitis virus, where defective interfering viral genomes (DVGs) accumulate during replication and skew the genome-to-PFU ratio (28, 29). Alternatively, LASV genomic RNA could be released in non-virion forms, such as extracellular vesicles, a phenomenon reported for zika virus and other flaviviruses (30, 31). In contrast, the coordinated decrease of both viral RNA and infectious titers observed for MOPV suggests a more effective cell-intrinsic restriction, potentially mediated by classical interferon responses, which have been shown to inhibit replication of various RNA viruses in macrophages (32). Together, these patterns likely reflect distinct equilibrium states between viral replication and innate restriction rather than differences in pathogenic potential and the distinct infection kinetics emphasize the relevance of Kupffer cells as a platform to dissect virus-specific immune control and tolerance mechanisms in reservoir hosts.

This information demonstrates the importance and necessity of further studies to better understand the process of viral clearance on the liver of Natal mice. In the context of hepatitis B virus (HBV), a model was described where Kupffer cells play key role in CD8 T cell-mediated viral clearance by which Kupffer cells increase the local hepatic CD8-positive T cell response to HBV in the liver in an IL-2R-dependent manner (7, 8). A similar mechanism could explain the control of infection in the tissue, without controlling the viral replication at a Kupffer cell level. Further immune characterization of infected Kupffer cells would be needed to answer these questions.

In summary, the development of the protocol described here represents a significant step forward in enabling the study of Kupffer cells and their immune functions in non-model species. By relying on the need of only one cross-reacting reagent, namely M-CSF, this method is adaptable to other species, broadening its potential applications to important zoonotic reservoirs like bats. Moreover, its minimally invasive design leaves other liver tissues largely undisturbed, aligning with 3R principles and supporting ethical research practices. This approach opens new possibilities for investigating immune processes within the liver microenvironment of the Natal mouse, providing insights into viral clearance mechanisms and apathogenic responses in natural reservoirs, with implications for future comparative immunological studies.

## Data Availability

The raw data supporting the conclusions of this article will be made available by the authors, without undue reservation.
